# Detection of hypoxia by near-infrared spectroscopy and pulse oximetry: a comparative study

**DOI:** 10.1117/1.JBO.27.7.077001

**Published:** 2022-07-25

**Authors:** Amanda Cheung, Lorna Tu, Andrew Macnab, Brian K. Kwon, Babak Shadgan

**Affiliations:** a University of British Columbia, International Collaboration on Repair Discoveries, Vancouver, British Columbia, Canada; b University of British Columbia, Departments of Pediatrics and Urologic Sciences, Vancouver, British Columbia, Canada; c University of British Columbia, Department of Orthopaedics, Vancouver, British Columbia, Canada; d University of British Columbia, School of Biomedical Engineering, Vancouver, British Columbia, Canada

**Keywords:** near-infrared spectroscopy, pulse oximetry, photoplethysmography, hypoxia, tissue oxygenation

## Abstract

**Significance:**

Pulse oximetry is widely used in clinical practice to monitor changes in arterial oxygen saturation (${\mathrm{SpO}}_{2}$). However, decreases in ${\mathrm{SpO}}_{2}$ can be delayed relative to the actual clinical event, and near-infrared spectroscopy (NIRS) may detect alterations in oxygenation earlier than pulse oximetry, as shown in previous cerebral oxygenation monitoring studies.

**Aim:**

We aim to compare the response of transcutaneous muscle NIRS measures of the tissue saturation index with pulse oximetry ${\mathrm{SpO}}_{2}$ during hypoxia.

**Approach:**

Episodes of acute hypoxia were induced in nine anesthetized Yucatan miniature pigs. A standard pulse oximeter was attached to the ear of the animal, and a transcutaneous NIRS sensor was placed on the hind limb muscle. Hypoxia was induced by detaching the ventilator from the animal and reattaching it once the pulse oximeter reported 70% ${\mathrm{SpO}}_{2}$.

**Results:**

Twenty-four episodes of acute hypoxia were analyzed. Upon the start of hypoxia, the transcutaneous NIRS measures changed in 5.3±0.4  s, whereas the pulse oximetry measures changed in 14.9±1.0  s (p<0.0001).

**Conclusions:**

Transcutaneous muscle NIRS can detect the effects of hypoxia significantly sooner than pulse oximetry in the Yucatan miniature pig. A transcutaneous NIRS sensor may be used as an earlier detector of oxygen saturation changes in the clinical setting than the standard pulse oximeter.

## 1 Introduction

Pulse oximetry is a simple, portable, and noninvasive tool widely used in clinical practice to evaluate the oxygenation status of a patient by monitoring arterial oxygen saturation (${\mathrm{SpO}}_{2}$).^1^^,^^2^ Each pulse oximeter unit consists of a monitor and a sensor. The monitor component controls light signal generation. The sensor component consists of one light-emitting diode (LED) that uses two red (600 to 750 nm) and near-infrared (NIR) (850 to 1000 nm) wavelengths to penetrate the skin and tissue, a photodetector (PD) to detect the light absorption, and a photon-counting processor.^3^ The optical technology is based on photoplethysmography (PPG), which is used to measure blood volume changes in the microvascular tissue bed. PPG detects the increase in light absorption during the systolic increase in arterial blood volume.^4^ Pulse oximeters measure the difference in light absorption of oxygenated ($O_{2}\mathrm{Hb}$) and deoxygenated hemoglobin (HHb) in pulsatile arterial blood to estimate the percentage of oxygen saturation in the arterial circulation.^5^^–^^7^

In situations in which a patient has poor peripheral perfusion, a pulse oximeter can be less reliable due to the pulse wave being inadequate for measurement.^7^ Similarly, pulse oximeters may lose their accuracy in patients with cardiac arrhythmia and will not measure at all following cardiac arrest when peripheral pulses are absent.^8^ Hypothermia, hypotension, or cold extremities may also contribute to the failure of an oximeter to register a signal.^9^ Movement artifacts are another factor that can affect the reading of ${\mathrm{SpO}}_{2}$.^10^^,^^11^ Abnormal hemoglobin levels as a result of the presence of carboxyhemoglobin or methemoglobin have also resulted in errors in ${\mathrm{SpO}}_{2}$ readings in both animal studies and clinical case reports.^12^^–^^15^ Other factors can also affect the reliability of pulse oximetry, such as fluorescent lighting and nail varnish.^16^^–^^18^ In addition, ${\mathrm{SpO}}_{2}$ is an estimate of arterial oxygen saturation (${\mathrm{SaO}}_{2}$) derived by arterial blood gas analysis with measures of ${\mathrm{SpO}}_{2}$ demonstrating a typical difference of ≤2% from ${\mathrm{SaO}}_{2}$.^19^^–^^21^ These differences are reported in subjects with ${\mathrm{SaO}}_{2}$ levels over 80%, so it is important to consider that the performance of pulse oximeters is less reliable when ${\mathrm{SaO}}_{2}$ is < 80%.^8^^,^^20^

Several studies have reported that decreases in ${\mathrm{SpO}}_{2}$ detected by pulse oximetry may lag behind the actual clinical event.^6^^,^^7^^,^^22^^,^^23^ We have also observed, when using different pulse oximeter systems in past animal experiments, that the onset of changes in ${\mathrm{SpO}}_{2}$ in response to acutely induced hypoxia can occur after significant delays. Being able to detect a critical cardiorespiratory event earlier can provide additional time for therapeutic interventions able to improve immediate care and outcome. For example, ${\mathrm{SpO}}_{2}$ is monitored in sedative dentistry to detect potential airway obstruction, and identifying hypoxia even seconds earlier would be vital and critical to restoring airway patency.^24^

Previous studies have demonstrated that cerebral oxygenation monitoring using near-infrared spectroscopy (NIRS) can detect alterations in oxygenation earlier than pulse oximetry.^25^^–^^29^ NIRS is a noninvasive optical technology used to measure tissue oxygenation and hemodynamics in various tissues, such as the brain, skeletal muscle, and spinal cord.^30^^–^^33^ Using similar physics principles to pulse oximetry, NIRS generates NIR light signals that penetrate tissue and measure the amount of light absorbed by tissue chromophores.^34^ With the use of a single light source with at least two wavelengths in the NIR spectrum (650 to 1000 nm) and one PD, NIRS is able to monitor changes in tissue oxygen delivery and consumption by measuring $O_{2}\mathrm{Hb}$, HHb, and the oxygenated–deoxygenated hemoglobin difference ($\mathrm{Hbdiff}=O_{2}\mathrm{Hb}-\mathrm{HHb}$), a relative measure of tissue oxygenation.^35^^–^^38^ In a multi-distance spatially resolved (SR) configuration (one PD with multiple light sources) or a single-distance configuration (one PD with one light source emitting multiple wavelengths), the light sources and PDs are adjacent to each other, which allows for calculation of the differential pathlength factor, the average distance a photon travels between the source and detector through tissue.^39^ This factor is incorporated into an algorithm derived from the modified Beer–Lambert law, which allows for an absolute measure of tissue oxygenation to be derived, providing relative changes in tissue oxygenation expressed as the tissue saturation index (TSI).^39^^–^^41^

The NIRS measures of tissue oxygen saturation are derived from a combination of blood in the arteries (25%), capillaries (5%), and veins (70%).^42^^,^^43^ The main difference between pulse oximetry and NIRS in terms of the calculations of oxygen saturation is the tissue being sampled. Pulse oximetry calculates the percentage of $O_{2}\mathrm{Hb}$ in arterial blood, whereas NIRS calculates the percentage of $O_{2}\mathrm{Hb}$ in both arterial and venous blood compartments.^21^

NIRS oxygenation monitoring is an already established approach in the management of traumatic brain injury to minimize hypoxic and ischemic brain damage.^44^ Several studies have reported reduced mortality and improved clinical outcomes from clinical decisions and diagnoses driven by NIRS monitoring.^45^^–^^47^ Patients benefiting from this approach include children on cardiopulmonary bypass,^48^^,^^49^ older adults during abdominal and orthopedic surgery who showed reduced postoperative cognitive impairment,^50^ and patients undergoing coronary artery bypass grafting with significantly shorter lengths of stay in the intensive care unit and postoperative hospitalization.^51^ Cerebral ischemia caused by technical issues with cannulation or cardiopulmonary bypass can also be detected in real time via NIRS monitoring.^50^^,^^52^ These studies emphasize the benefit and importance of NIRS monitoring as a means of maintaining an adequate oxygen supply or rapidly restoring interrupted oxygen delivery. As NIRS does not require pulsatile blood flow, it can be a valuable tool in situations in which pulse oximetry has limited applicability, e.g., in periods of low blood flow occurring in hypotensive patients, during cardiopulmonary bypass, and when the circulation is nonpulsatile, such as cardiac arrest. To our knowledge, there are no reports to date comparing transcutaneous NIRS monitoring to pulse oximetry during acute hypoxia, as previous comparative studies focused on cerebral NIRS measurements.^25^^–^^29^ Transcutaneous NIRS monitoring of tissue hypoxia provides a better alternative to cerebral NIRS monitoring as it uses a simple and small sensor that can be easily placed on any part of the body over the skin. Cerebral NIRS systems require a large sensor with a 3- to 4-cm interoptode distance (IOD) because a higher depth of penetration is needed to access the brain cortex, whereas a transcutaneous NIRS system can use a smaller sensor with a 1-cm IOD. It would be highly advantageous when limited locations are available for monitoring.

This study aimed to compare transcutaneous muscle NIRS and pulse oximetry in detecting the onset of hypoxia. Specifically, we compared the time of onset between changes in hind limb muscle oxygenation measured with NIRS to changes in arterial oxygen saturation measured with pulse oximetry in anesthetized Yucatan miniature pigs during induced acute hypoxia. The NIRS sensor was placed on the thigh muscle of the animal, a standard and common region for NIRS measurements. At the same time, the pulse oximeter was attached to the ear of the animal, a standard placement for pulse oximetry. Because the application of pulse oximetry on the hind limb is not standard practice and we could not place our NIRS sensor on the ear, we did not compare NIRS and pulse oximeter readings from the same region. Still, we compared two standard and applicable regions between the two different techniques.

## 2 Material and Methods

All animal protocols and procedures performed in this study were approved by the Animal Care Committee of the University of British Columbia (UBC) and were compliant with the policies of the Canadian Council of Animal Care, the U.S. Army Medical Research and Materiel Command (USAMRMC), and the Animal Care and Use Review Office (ACURO). The anesthesia/analgesia protocols were established by the UBC Center for Comparative Medicine.

### 2.1 Porcine Model and Transcutaneous NIRS Sensor

Nine female Yucatan minipigs weighing 25 to 31 kg from previous studies that employed NIRS for investigating spinal cord hemodynamics after spinal cord injury (SCI) were analyzed.^33^ Animals were prepared for surgery, intubated, and anesthetized as previously described.^53^^,^^54^ In brief, animals were anesthetized with intramuscular telazol (5 mg/kg) and xylazine (1 mg/kg) and then endotracheally intubated. Propofol (2 mg/kg), fentanyl (10  μg/kg), and ketamine (11 mg/kg) were used for anesthesia induction, and propofol (8 mg/kg/h), fentanyl (12  μg/kg/h), and ketamine (11 mg/kg/h) were used for anesthesia maintenance through a continuous rate infusion. Animals were mechanically ventilated with a ventilator rate of 12 breaths/min and a tidal volume of 12 to 15 mL/kg with 1.4 L (70%) nitrogen and 0.6 L (30%) of oxygen (Veterinary Anesthesia Ventilator model 2002, Hallowell EMC, Pittsfield, Massachusetts). Standard monitoring of the animals was performed throughout the procedure, including monitoring of the respiratory rate, blood pressure, end-tidal carbon dioxide, heart rate, and oxygen saturation.

**Fig. 1:**
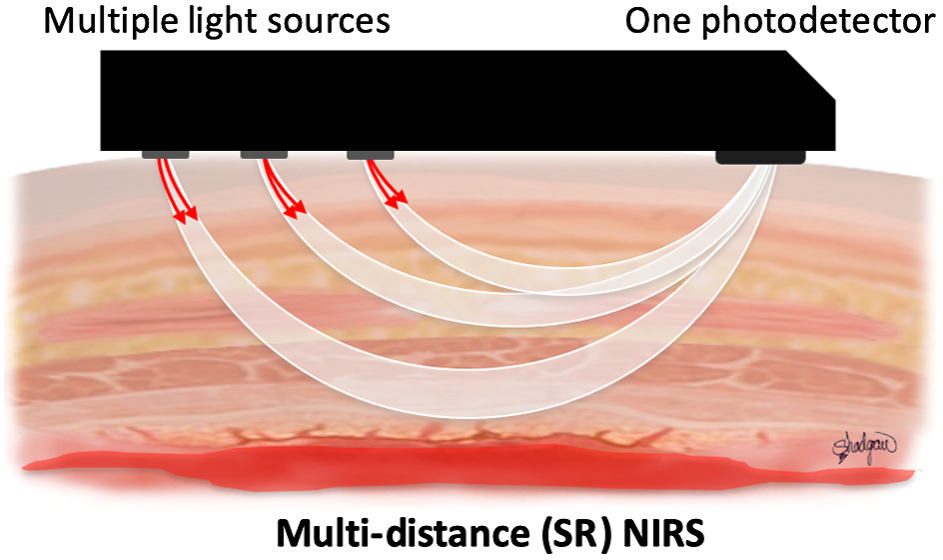
A multi-distance SR NIRS sensor.

A standard pulse oximeter system was attached to the ear of the animal to measure arterial oxygen saturation and heart rate at a frequency of 0.25 Hz (Rad-5 Masimo, California). A transcutaneous NIRS sensor (PortaMon, Artinis Medical Systems, Elst, The Netherlands) was placed on the biceps femoris muscle belly of the hind limb of the animal. The NIRS system uses a continuous-wave multi-distance SR NIRS configuration (Fig. 1) and an NIRS algorithm based on the modified Beer–Lambert law to measure changes in skeletal muscle $O_{2}\mathrm{Hb}$ and HHb concentrations and calculate TSI% at a sampling rate of 10 Hz.^55^ The sensor has three LEDs with two wavelengths (760 and 850 nm) and one photodiode with ambient light protection. The distances between the emitters and the PD are 30, 35, and 40 mm, and the sensor size is 83.8×42.9×17.2  mm.

The software of the NIRS sensor (Oxysoft, Artinis Medical Systems, Elst, The Netherlands) uses an SR spectroscopy algorithm that measures the slope of the optical density change as a function of multiple distances. This method enables the calculation of scaled absorption coefficient measures, which can allow for measuring values of chromophore ratios and calculating tissue oxygen saturation index in percentage.^39^

Episodes of acute hypoxia were performed in each animal. Hypoxia was induced by disconnecting the animal from the ventilator and reattaching it once ${\mathrm{SpO}}_{2}$ reached up to 70% SpO_2,_ as identified by the pulse oximeter. The target of 70% ${\mathrm{SpO}}_{2}$ was selected to induce a severe hypoxic response related to the spinal cord hemodynamics study that was primarily investigated in animals.^33^

### 2.2 Data Analysis

The first detectable change in the signals was calculated by determining when the value of ${\mathrm{SpO}}_{2}$ and TSI first changed from their respective baseline values after induction of hypoxia. For the pulse oximeter, the time needed for the ${\mathrm{SpO}}_{2}$ value to change on the monitor from its baseline value once hypoxia was induced was noted. For the NIRS signal, the data trace was analyzed, and the time point in which the TSI value changed from its baseline value was noted. Comparisons between the time when the first detectable change was seen in the NIRS and pulse oximetry measurements were calculated using the Student’s t-test, with the level of significance set at p<0.05. This comparison was also calculated after downsampling the NIRS data from 10 to 0.25 Hz to compare the NIRS and pulse oximetry data when sampling at the same frequency. Data were analyzed using GraphPad Prism 9.0.0 (GraphPad Software, La Jolla, California). Data are presented as mean ± standard error of the mean.

## 3 Results

We analyzed 24 episodes of acute hypoxia in nine female Yucatan miniature pigs. Hypoxia was induced and allowed to proceed with anticipated drops in ${\mathrm{SpO}}_{2}$ up to 70%.

### 3.1 Comparison of SpO_2_ and TSI Detection Times During Hypoxia

**Fig. 2:**
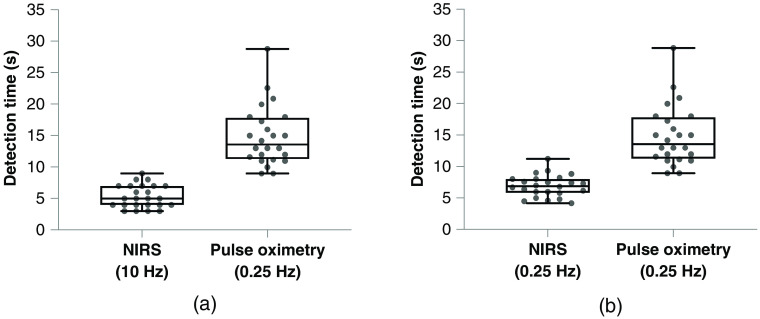
Comparison of the detection times for the first detectable change in oxygen saturation measured by NIRS TSI sampling at (a) 10 Hz and (b) 0.25 Hz with pulse oximetry arterial oxygen saturation (${\mathrm{SpO}}_{2}$) sampling at 0.25 Hz upon the onset of acute hypoxia.

For all 24 hypoxic episodes combined, the first detectable change in NIRS TSI (sampling at 10 Hz) occurred in 5.3±0.4  s, and the first detectable change in ${\mathrm{SpO}}_{2}$ occurred in 14.9±1.0s (p<0.0001) [Fig. 2(a)]. The NIRS data were then downsampled to 0.25 Hz to compare the NIRS data with the same pulse oximetry data sampling frequency. The first detectable change in NIRS TSI (sampling at 0.25 Hz) occurred in 6.9±0.3  s compared with the first detectable change in ${\mathrm{SpO}}_{2}$ occurring in 14.9±1.0s (p<0.0001) [Fig. 2(b)]. In all cases, the transcutaneous NIRS sensor detected changes in oxygen saturation earlier than the pulse oximeter.

### 3.2 Changes in TSI Magnitude During Hypoxia

**Fig. 3:**
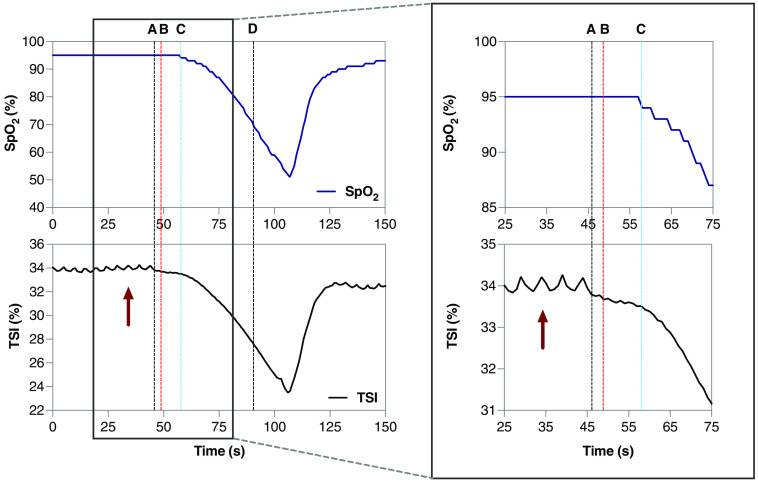
Changes in arterial oxygen saturation (${\mathrm{SpO}}_{2}$) and TSI during acute hypoxia in one animal. The dotted black line at A and D represents when the ventilator was (A) detached and (D) reattached. The dotted red line at B represents when the first change in TSI was detected. The dotted blue line at C represents when the first change in ${\mathrm{SpO}}_{2}$ was detected. Red arrows show respiration cycles detected by the NIRS sensor; note the absence of respiratory cycles during hypoxia induction.

During acute hypoxia, the TSI decreased on average by 10.5±1% from a baseline of 70.6±7.6%. A representative graph showing the onset of changes between ${\mathrm{SpO}}_{2}$ (sampling at 0.25 Hz) and TSI (sampling at 10 Hz) is shown in Fig. 3. The pulse oximeter and transcutaneous NIRS sensor showed a similar pattern of change during acute hypoxia, depicted by a sharp decrease in their respective oxygenation measurement and followed by an immediate increase as the animal recovered.

### 3.3 Effect of Ventilation on NIRS Signals

The transcutaneous NIRS sensor recorded regular $O_{2}\mathrm{Hb}$ and TSI oscillations with a frequency of 0.2 Hz during the experiment. The rhythm of regular oscillations in all animals that were connected to the ventilator was similar, and the respiratory rate was set at 12 breaths/min. The oscillations disappeared during the hypoxia episodes (Fig. 3).

## 4 Discussion

In this study, we demonstrated that NIRS-derived TSI% responds in 5.3±0.4s when sampling at 10 Hz and 6.9±0.3s when sampling at 0.25 Hz (the same sampling frequency as the pulse oximeter) upon the start of acute hypoxia, whereas the pulse oximeter ${\mathrm{SpO}}_{2}$ responds in 14.9±1.0s. Our results show that transcutaneous NIRS can detect the effects of hypoxia earlier than pulse oximetry and transcutaneous NIRS can detect and monitor respiratory cycles in real time.

Pulse oximetry measurements may lag behind a change in the patient’s oxygenation status and present a delay in detecting hypoxic events. These delays may be a result of irregular pulse volume or rhythm, which can slow down the instrument’s response, or a result of computation delay due to the signal averaging algorithms in the device.^56^ Individual pulse oximeters have varying specifications and hence different operating characteristics, and the signal averaging required to derive the percentage value displayed always takes several seconds, and in some instruments, it can even exceed 20 s.^7^^,^^20^ Due to such a signal lag, there is a period during which the actual arterial blood oxygen saturation starts to fall in real time before a measurement is completed and displayed by the pulse oximeter. It is important for this limitation to be recognized and, in particular, for clinicians and nurses to be aware of this time delay between the beginning of potentially critical hypoxia and the reflection of the event in its ${\mathrm{SpO}}_{2}$ reading. Here, NIRS is advantageous as it can monitor respiration and detect hypoxia sooner than pulse oximetry by operating at higher frequencies.

Previous studies centered on cerebral NIRS oxygenation measurements in comparison with pulse oximetry during hypoxic events. In 2007, Tobias reported that hypercyanotic spells during anesthesia resulted in a decrease in cerebral oxygenation [regional oxygen saturation (${\mathrm{rSO}}_{2}$)] monitored by NIRS 15 to 30 s prior to any change in pulse oximetry.^25^ In 2008, Tobias found that a 5% decrease in ${\mathrm{rSO}}_{2}$ in 10 patients occurred 52 s earlier than a similar decrease in oxygenation measured by pulse oximetry.^26^ Studies in pediatric patients have shown a delay of ${\mathrm{SpO}}_{2}$ compared to NIRS ${\mathrm{rSO}}_{2}$ during hypoxia^10^^–^^12^ and that ${\mathrm{rSO}}_{2}$ decreased about 40 s earlier than ${\mathrm{SpO}}_{2}$ during hypoxia generated by paused mechanical ventilation.^27^ In this study, we found that, similar to cerebral oxygenation, muscle oxygenation monitored by NIRS decreased about 10 s before any changes were detected by pulse oximetry upon the start of acute hypoxia. NIRS TSI responded in 5.3±0.4  s whereas the pulse oximeter responded in 14.9±1.0  s. Similarly, with the NIRS and pulse oximeter monitoring at the same frequency (0.25 Hz), NIRS detects changes in oxygenation about 8 s earlier than pulse oximetry, with NIRS TSI% responding in 6.9±0.3  s and the pulse oximeter taking 14.9±1.0  s to show a drop in ${\mathrm{SpO}}_{2}$.

We recognize limitations in what we report, especially because we are comparing different technologies applied to two different anatomical locations. The ear is a common place for transmission pulse oximetry, whereas the hind limb is a common region for NIRS monitoring. We are unable to monitor ${\mathrm{SpO}}_{2}$ on the hind limb using conventional pulse oximeters, and applying conventional NIRS sensors such as the Artinis PortaMon used in this study or even the smaller Artinis PortaLite-mini (size: 40×18×5  mm) on the ear lobe is not possible. Therefore, we used two different standard regions to compare the sensitivity of these two technologies that monitor transcutaneous oxygenation changes to detect the onset of acute hypoxia. In addition, we acknowledge that the oxygen saturation we measured in the ear with pulse oximetry was a different location from what we measured in the hind limb with NIRS, but this regional difference did not by itself explain the differences in the measurements that we report in this study. Arterial oxygen saturation is not tissue-dependent as it indicates the amount of oxygen within the arterial stream that supplies each organ and tissue. However, tissue oxygen saturation is a combination of arterial and venous streams at the microvascular level that can be different in different regions based on their local metabolism and hemodynamics. In this context, Tobias suggested that the different hypoxia detection times between NIRS and pulse oximetry are related to the vascular compartments that the technologies are evaluating. During hypoxia, the partial pressure of oxygen decreases at an equal rate in venous and arterial blood; however, given the lower venous partial pressure of oxygen, it decreases more rapidly on the oxyhemoglobin dissociation curve, and a decrease in NIRS tissue oxygenation would occur first.^26^ Therefore, the difference in detection times is due to the sensitivity to different vascular compartments, in which pulse oximetry monitors arterial oxygen saturation and NIRS reflects changes in arterial, capillary, and venous saturation. We recognize that different sampling frequencies between the NIRS sensor and pulse oximeter may also contribute to the observed time-delay differences. The time difference between 10 and 0.25 Hz sampling rates for the NIRS data was 1.6±0.2  s. In addition, the animals that we used for this study were part of a larger study exploring the local effects of SCI on cord hemodynamics, and the episodes of acute hypoxia were studied both prior to the cord injury occurring and 7 days after the animals had experienced an acute traumatic SCI. There is also the potential limitation that there is no absolute baseline TSI value; hence each patient’s resting value serves as their baseline. However, both sensors monitor transcutaneous oxygenation changes and provide a comparable measurement to allow us to assess whether NIRS is more sensitive than pulse oximetry in detecting oxygenation changes.

A clinical setting where our findings that transcutaneous NIRS data have advantages over pulse oximetry is relevant in sedative dentistry, in which hypoxia is a critical problem.^57^ With the use of conscious sedation techniques, it is standard to monitor ${\mathrm{SpO}}_{2}$ to detect potential oral cavity or airway obstruction as this is a clinical situation that can become critical in seconds if its onset is not recognized and patency of the airway is not restored.^24^ With the ability of NIRS to detect changes in oxygenation earlier than pulse oximetry and monitor patients regardless of blood pressure fluctuation and variations in peripheral perfusion, the advantages of monitoring oxygenation using NIRS would appear to provide additional safety for patients during dental procedures requiring conscious sedation. Furthermore, the ability to monitor the respiratory rate and rhythm in addition to tissue oxygen saturation highlights potential applications of transcutaneous NIRS for monitoring cardiorespiratory function at the bedside.^15^

Overall, our study provides new evidence of the ability of transcutaneous muscle NIRS to detect the effects of hypoxia earlier than pulse oximetry. Hence, the use of a transcutaneous muscle NIRS sensor in many clinical situations may be a more sensitive and earlier detector of systemic hypoxia than the standard pulse oximeter.
